# Theoretical Investigations of Optical Origins of Fluorescent Graphene Quantum Dots

**DOI:** 10.1038/srep24850

**Published:** 2016-04-20

**Authors:** Jingang Wang, Shuo Cao, Yong Ding, Fengcai Ma, Wengang Lu, Mengtao Sun

**Affiliations:** 1 Department of Chemistry and Department of Physics, Liaoning University, Shenyang 110036, PR China; 2 Beijing National Laboratory for Condensed Matter Physics, Beijing Key Laboratory for Nanomaterials and Nanodevices, Institute of Physics, Chinese Academy of Science, Beijing, 100190, P. R. China; 3 Department of Physics, Shenyang Aerospace University, 110036, PR China

## Abstract

The optical properties of graphene quantum dots (GQDs) were investigated theoretically. We focused on the photoinduced charge transfer and electron-hole coherence of single-layer graphene in the electronic transitions in the visible regions. Surface functionalization with donor or acceptor groups produced a red shift in the absorption spectrum, and electrons and holes were highly delocalized. The recombination of excited, well-separated electron-hole (e–h) pairs can result in enhanced fluorescence. This fluorescence enhancement by surface functionalization occurs because of the decreased symmetry of the graphene resulting from the roughened structure of the surface-functionalized GQDs.

Graphene is a single atomic layer that consists of a two-dimensional honeycomb lattice of carbon atoms. Because of its fundamental physics and excellent optical, thermal and electrical properties, it has been widely investigated, both experimentally and theoretically^1^,^2^,^3^,^4^. Graphene has been widely applied in quantum information processing^5^, fuel cells^6^, photovoltaic materials^7^, water purification^8^, controlling reactions^9^, and optical modulation^10^.

Because the fragments of graphene are limited in size or domains, graphene quantum dots (GQDs) are emerging as an advanced material with multiple functionalities because of their unique optical, electronic^11^, spin^12^, and photoelectric properties induced by edge effects and quantum confinement effects. There are many important applications for GQDs in fluorescent materials^13^, photocatalysis^14^, bioimaging^15^ and organic photovoltaic (OPV) solar cells^16^. To apply GQDs to optoelectronic nano devices, elucidating and understanding the mechanisms underlying their novel light-absorption/emission properties is of critical importance. The influence of edge structure on the electronic properties of GQDs with 2–20-nm lateral dimensions has been investigated^11^. When the size of a GQD is less than 10 nm, quantum confinement and edge effects can induce photoluminescence (PL)^17^,^18^. Many efforts have been directed at increasing the quantum yield of GQDs, for example, by surface functionalization of the GQDs (SF-GQDs) with organic molecules^19^ or surface oxidation^20^. The PL of GQDs can be tuned based on the charge-transfer effect of electron-withdrawing or electron-donating functional groups^21^. The PL mechanism of GQDs has been interpreted as the minimization of thermalization resulting from electron-phonon scattering^22^, or the formation of an excited-state relaxation channel, which causes inelastic light scattering by electric doping^23^. Kim attributed this behavior to fast carrier–carrier scattering^24^, which encourages the direct recombination of the excited electron–hole (e–h) pairs that produce this PL before the carriers are thermalized in the lattice.

In this paper, we investigated the effects of surface functionalization on the optical properties of GQDs with a lateral dimension of approximately 2 nm, mainly focusing on the charge-transfer and e–h coherence in the electronic transition of GQDs. We also evaluated the influence of the geometrical roughness resulting from surface functionalization with organic molecules on the GQDs’ optical properties. Our results can facilitate a deeper understanding of the origin of the optical properties of GQDs.

## Results

**Figure 1:**
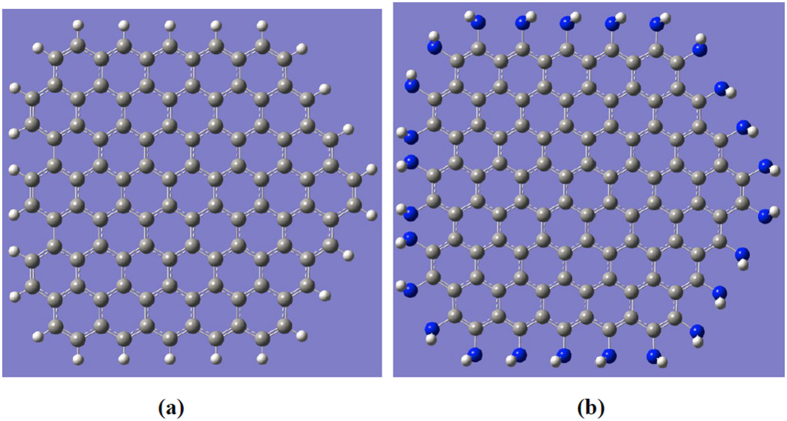
The models used for the calculations. (**a**) GQD and (**b**) SF-GQD with -NH_2_.

**Figure 2:**
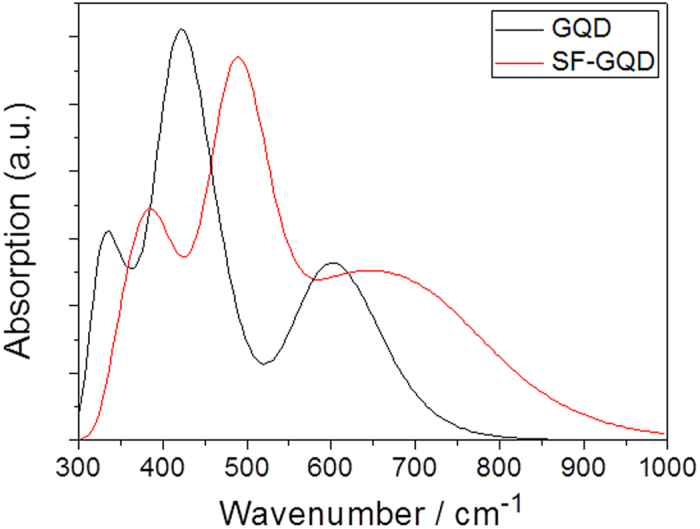
The calculated absorption spectra of the GQD and SF-GQD with -NH_2_.

The models used for the calculations can be seen in Fig. 1(a,b), which show a GQD and an SF-GQD with -NH_2_, respectively. In mode (a), there are four zigzag edges and one armchair edge. The calculated absorption spectra of both GQDs in the visible region are presented in Fig. 2 and demonstrate that the optical absorption peaks of SF-GQD with -NH_2_ groups are significantly red shifted. This finding indicated that the fluorescence peaks of SF-GQD with -NH_2_ should also be significantly red shifted. These red-shift phenomena are consistent with the experimental findings^13^,^19^,^21^.

**Figure 3:**
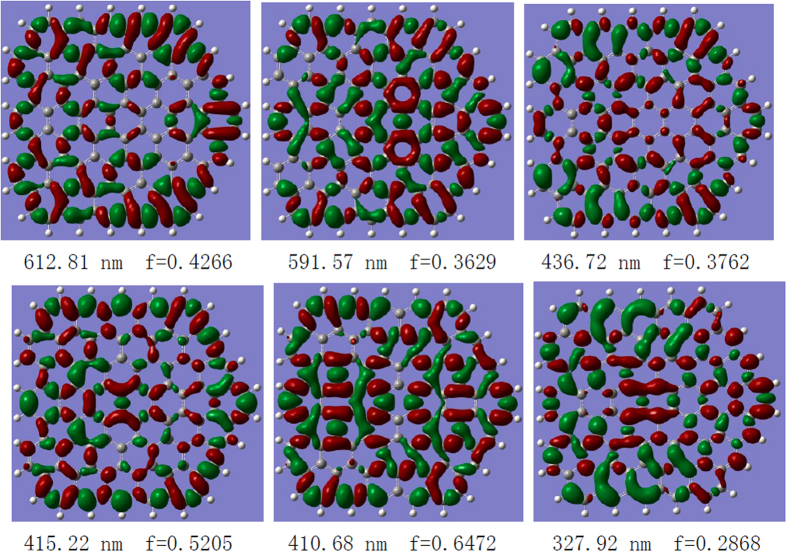
Charge-transfer densities for the strong electronic transitions in the GQD, where the holes and electrons are represented in green and red, respectively.

To reveal the nature of the optical origins of fluorescent GQDs, it is important to observe the e–h coherence and electron transfer in electronic transitions in the visible region. The degree of electron transfer determines the degree of fluorescence because the direct recombination of excited e–h pairs produces fluorescence^24^. We first demonstrate the degree and orientation of charge transfer for GQDs for strong electronic transitions. The charge difference densities (CDDs) in Fig. 3 reveal that the electrons and holes were locally excited and that the electrons transferred from the adjacent holes. Therefore, the electrons and holes were not significantly separated, and the degree of electron transfer from holes is weak because of the strong interaction between holes and electrons in the electronic transitions in the visible region. Note that electrons seem to significantly transfer from holes in the near-ultraviolet region (see CDD at 327.9 nm) but cannot contribute to the fluorescence in the visible region.

The three-dimensional CDDs in Fig. 3 demonstrate the qualitative visualization analysis of photoinduced charge transfer. To quantitatively analyze the charge transfer, Δ*r* is introduced to measure the charge-transfer length^25^,


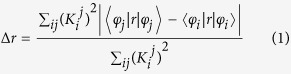


where i and j traverse all of the occupied and virtual molecular orbitals, respectively, and φ is the orbital wave function. When Δ*r* > 2, the electronic state transition is considered in the charge-transfer excited state, whereas when Δ*r* < 2.0, it is considered to be in an excited state with a different charge^25^. The calculated results are summarized in Table 1, where the electronic transition states of GQDs are localized excited states, and the electronic state transitions of SF-GQD are the charge-transfer excited states.

**Figure 4:**
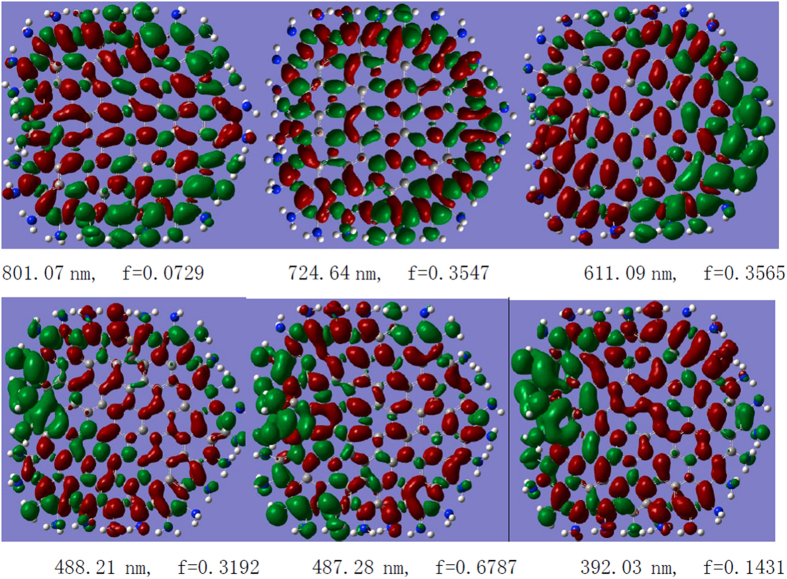
Charge-transfer densities for the strong electronic transitions in the SF-GQD with -NH_2_, where the holes and electrons are represented in green and red, respectively.

Therefore, all of the strong electronic transitions of-GQDs are charge-transfer excited states because the charge-transfer lengths of these excited states all exceed 2.0 Å. The CDDs of these excited states confirm this phenomenon; the holes and electrons are well separated, and the degree and orientation of the electron transfer are well visualized (Fig. 4). For example, for the electronic transition at 801 nm, almost all holes are localized on the edge, whereas the electrons are localized on the center. For the electronic transition at 611 nm, the holes are localized on the right edges, and the electrons are delocalized to the center of graphene and the left edges. This behavior means that in this excited state, the optical excitation occurs at the right edge, and then the electrons excited at this edge are delocalized to the other parts, indicating strong electron transfer in the excited state.

## Discussion

**Figure 5:**
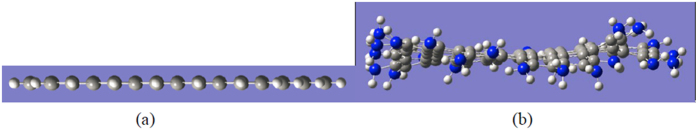
Optimized molecular structures. (**a**) GQD and (**b**) SF-GQD with -NH_2_.

The surface functionalization of GQDs can result in a red shift in the absorption spectrum and a corresponding red shift in the fluorescence spectrum. Our theoretical calculations are consistent with the experimental results. The CDDs in Fig. 4 reveal that the mechanism of fluorescence enhancement is not, as expected, mainly attributable to the functionalized donor or acceptor groups because both electrons and holes exist on the functionalized -NH_2_. To further reveal the mechanism of fluorescence enhancement by surface functionalization, we also obtained the optimized molecular geometries of GQDs and SF-GQDs with -NH_2_ (see Fig. 5). The optimized GQD geometry was found to be flat, whereas the optimized F-GQD geometry was not highly flattened, thereby decreasing the symmetry of the graphene. Therefore, the holes can be localized on the edge of the SF-GQD, and the electrons can transfer to other parts.

We visualized the theoretical mechanism of fluorescence enhancement in surface-functionalized GQDs using CDD. This surface functionalization can result in a red shift in the absorption spectrum and roughness in the graphene structure. The decrease in the GQD’s symmetry by surface functionalization also causes the electrons to delocalize along the graphene. The large electron transfer and well-separated e–h pairs can significantly enhance the fluorescence because the direct recombination of excited e–h pairs produces strong fluorescence. Our results can promote a deeper understanding of the origin of the optical properties of GQDs. It should be noted that the doping, size and edge are also important for the fluorescence enhancement. They will be studied in our further theoretical work.

## Methods

The models used in the calculations are shown in Fig. 1(a,b): graphene and surface-functionalized graphene with -NH_2_, respectively. In mode (a), there are four zigzag edges and one armchair edge. All the calculations were performed with Gaussian 09 software^26^. The ground-state geometries of both GQDs were optimized with density functional theory (DFT)^27^ using the B3LYP functional^28^ and 6-31g(d) basis set. Their optical absorption spectra in the visible region were calculated with time-dependent DFT (TD-DFT)^29^ using the CAM-B3LYP functional^30^ and 6-31g(d) basis set. Note that the long-range-corrected functional (CAM-B3LYP) was employed for the non-Coulomb part of the exchange functional. The charge transfer in the electronic transition was visualized using CDDs^31^.

## Additional Information

**How to cite this article**: Wang, J. *et al*. Theoretical Investigations of Optical Origins of Fluorescent Graphene Quantum Dots. *Sci. Rep.* **6**, 24850; doi: 10.1038/srep24850 (2016).

## Figures and Tables

**Table 1:** Charge-transfer lengths for the GQD and SF-GQD.

**GQD**			**SF-GQD**		
**nm**	**f**	**ΔL(A)**	**nm**	**f**	**ΔL(A)**
612	0.4266	**1.08**	803	0.0729	**2.10**
591	0.3629	**1.26**	724	0.3547	**2.95**
436	0.3762	**1.78**	611	0.3565	**3.44**
415	0.5205	**1.41**	488	0.3192	**3.59**
411	0.6472	**0.26.**	487	0.6787	**3.00**
328	0.2868	**1.64**	392	0.1431	**4.35**
